# HYPER IgE SYNDROME WITH UMBILICAL HERNIA

**DOI:** 10.4103/0019-5154.57619

**Published:** 2009

**Authors:** N C Hiremath, N T Madan Mohan, C Srinivas, Prabhakar M Sangolli, K Srinivas, N Soumya

**Affiliations:** *From the Department of Dermatology, Dr. B.R. Ambedkar Medical College and Hospital, K.G. Halli, Bangalore - 560 045, Karnataka, India.*

**Keywords:** *Hyper IgE syndrome*, *umbilical hernia*, *inguinal hernia*

## Abstract

Hyper IgE Syndrome (HIES) is a rare multi system genetic immunodeficiency disorder, with immunological and non-immunological features. Immunolgical features are 1) Recurrent cutaneous abscesses, 2) Atopic dermatitis like lesions, 3) Sino pulmonary infections, 4) Elevated serum IgE levels and 5) Abnormal neutrophil chemotaxis. Non immunological features include cranio facial and skeletal abnormalities. We are reporting a girl with classical features of HIES with umbilical hernia with her younger brother suffering from right sided inguinal hernia, as both herniae are hitherto unreported in patients with HIES.

## Introduction

First described in 1966, hyper immunoglobulin E syndrome (HIES) is a rare primary genetic immunodeficiency disorder with both autosomal dominant and autosomal recessive mode of inheritance.[1] It has two variants, namely: Job's syndrome and Buckley's syndrome. Autosomal recessive patients tend to have severe molluscum contagiosum and other viral infections and may develop severe neurological complications. These patients also lack skeletal or dental involvement and do not develop lung cysts.[2]

We are reporting of a girl with classical features of HIES with umbilical hernia. Her clinically normal younger brother had right-sided inguinal hernia.

## Case Report

A 5-year-old girl, offspring of a second-degree consanguinous marriage, presented with generalized itching and crusted lesions over the face and scalp of one month duration and swelling of face since three days. The patient had past history of recurrent pyoderma, abscesses, empyema thoracis, otitis media, and oral candidiasis with onset of illness when the patient was one-week old. There was no history of similar illness among family members. Her younger brother was operated for right-sided inguinal hernia. Clinically, the child was poorly built, febrile, with pallor, edema, and lymphadenopathy. She had bilateral basal crepitations and reducible umbilical hernia. On cutaneous examination, she had generalized crusted erosions, periorbital edema, and blepharo conjunctivitis with massive submandibular and submental lymphadenopathy. Clinical differential diagnosis of severe atopic dermatitis, HIES, Wiscott Aldrich syndrome, and X-linked a gamma globulinemia were considered. Investigations revealed neutrophilic leucocytosis and elevated ESR. Absolute eosinophil count (AEC) was 1920 cells/mcl (normal AEC = 150-450 cells/mcl). Pus for culture from incision and drainage site, skin lesions, and ear discharge yielded *Staphylococcus aureus*. X-ray chest demonstrated right lower lobe pneumonia. X-ray thoracolumbar spine was normal. Serum IgE was 1654IU/ml (normal 100IU/ml). X-ray knee joints showed valgus deformity.

Skin biopsy findings were hyperkeratotic epidermis with dermal perivascular mononuclear infiltrate. Final diagnosis of HIES was made as our patient satisfied all criteria for diagnosis of HIES. She was treated with IV anti-staphylococcal antibiotics, Condy's compresses, oral fluconazole, and povidone iodine shampoo. The child showed good improvement.

## Discussion

Our patient had all the characteristic features of HIES. However, *in vitro* neutrophil chemotaxis test to chemoattractants like formyl methionine, leucine, and phenyl alanine (fMet, leu, phe) could not be carried out as the patient could not afford it. Nonimmunological features present in our patient included coarse facies with wide inter alar base, pathological fracture of right fibula, genu valgum and umbilical hernia.

Osteopenia with pathological fractures are seen in 60% of patients with HIES.[3] Grimbacher *et al.*, reported genu valgum in three of their 30 patients of HIES.[4] Bochdalek hernia has been reported in Job's syndrome by Butterworth *et al*.[5] However, umbilical hernia has not been reported in HIES to date. These nonimmunolgical features are believed to be due to abnormal cytokine profile or mesenchymal cell defect leading to abnormal bone and connective tissue turnover.[6,7] Thus, the presence of umbilical hernia in a classical case of HIES with inguinal hernia in younger sibling prompted us to report this case.
